# Adaptive immune dysregulation drives new-onset refractory status epilepticus

**DOI:** 10.3389/fimmu.2026.1874730

**Published:** 2026-07-30

**Authors:** Zhi-Ying Phong, Si-Lei Fong, Kheng-Seang Lim, Suhailah Abdullah, Hong-Gee Lee, Ching-Soong Khoo, Chung Yeng Looi, Won Fen Wong

**Affiliations:** 1 Department of Medical Microbiology, Faculty of Medicine, Universiti Malaya, Kuala Lumpur, Malaysia; 2 Division of Neurology, Department of Medicine, Faculty of Medicine, Universiti Malaya, Kuala Lumpur, Malaysia; 3 Department of Clinical Pharmacy and Pharmacy Practice, Faculty of Pharmacy, Universiti Malaya, Kuala Lumpur, Malaysia; 4 Neurology Unit, Department of Medicine, Faculty of Medicine, Universiti Kebangsaan Malaysia, Kuala Lumpur, Malaysia; 5 School of Biosciences, Faculty of Health & Medical Sciences, Taylor’s University, Subang Jaya, Malaysia; 6 Department of Biotechnology, Faculty of Applied Sciences, UCSI Universitiy, Cheras, Malaysia

**Keywords:** autoimmune encephalitis (AIE), B cells, CAR T cell therapy, new-onset refractory status epilepticus (NORSE), NMDAR, T cells

## Abstract

New-onset refractory status epilepticus (NORSE) is a life-threatening neurological condition that emerges abruptly in individuals without a prior history of epilepsy and is characterised by seizures that are refractory to standard antiseizure medications. NORSE is associated with high mortality and long-term neurological deficits, and autoimmune encephalitis (AIE) represents a major identifiable cause of NORSE. Growing evidence implicates immune-mediated mechanisms as central drivers of NORSE; however, existing studies have focused predominantly on innate immune dysregulation, including cytokine-driven neuroinflammation and microglial activation, whilst the role of adaptive immune mechanisms in AIE-mediated NORSE remains relatively underexplored. This review synthesises current evidence linking adaptive immune responses in NORSE pathogenesis, with particular emphasis on B and T lymphocytes in shaping neuroinflammation. B cell–mediated response is implicated in NORSE cases associated with AIE where autoantibodies such as anti-N-methyl-D-aspartate receptor (NMDAR) disrupt neuronal signalling and contribute to seizure generation. Beyond humoral immunity, T-cell subsets, for instance, cytotoxic T cells, contribute to neuronal damage through recognition of neuronal intracellular antigens. Emerging immunomodulatory therapies targeting adaptive immune components are also discussed, including monoclonal antibody therapies and innovative approaches inspired by conventional chimeric antigen receptor (CAR) T-cell technology. A deeper understanding of the complex interplay between innate and adaptive immune responses is essential to elucidate disease mechanisms and guide future development of more precise mechanism-based therapeutic strategies for NORSE.

## 1 Introduction

Epilepsy is the second most prevalent neurological disorder worldwide, affecting approximately 50 million people. It is a chronic, noncommunicable condition characterised by recurrent seizures arising from an imbalance between excitatory and inhibitory neuronal activity in the brain, resulting in hypersynchronous and excessive electrical discharges that disrupt normal neuronal function (1). Status epilepticus (SE) is a life-threatening neurological emergency defined by prolonged seizure activity, including generalised tonic-clonic seizures lasting beyond 5 min or focal seizures exceeding 10 min (2). Management of SE requires a rapid, stepwise escalation. First-line treatment involves immediate administration of a benzodiazepine to achieve early seizure termination. If seizures persist, second-line therapy consists of an intravenous anti-seizure medication, such as levetiracetam. When seizures continue or recur despite these first- and second-line interventions, the condition is termed refractory status epilepticus (RSE) and necessitates third-line therapy with continuous intravenous anaesthetic agents. Persistent or recurrent seizure activity despite anaesthetic treatment is classified as super-refractory status epilepticus (SRSE).

New-onset refractory status epilepticus (NORSE) is neither a distinct diagnosis nor a clinical entity; rather, it is a terminology used to describe the clinical presentation. NORSE refers to a presentation in individuals without a prior history of epilepsy or other pre-existing relevant neurological disorder who develop new-onset RSE in the absence of a clear acute or active structural, toxic, or metabolic cause (3). A population-based study in Eastern Finland estimated the incidence rate of NORSE to be around 0.65 per 100,000 person-years (4). Febrile infection-related epilepsy syndrome (FIRES) is a subgroup of NORSE that is typically preceded by a prior febrile illness two weeks to 24h before the onset of seizure, with or without fever at the onset of SE (3). Mortality in NORSE and FIRES remains substantial, estimated at approximately 10% in children and up to 30% in adults (5–8). Among survivors, the outcomes are frequently devastating. A large proportion develop chronic, drug-resistant epilepsy, requiring long-term multidrug therapy with limited seizure control. In addition, many patients experience permanent neurocognitive impairment, including deficits in memory, attention, and executive function, often accompanied by behavioural and psychiatric disturbances. Functional independence is commonly compromised, which significantly impacts the quality of life.

NORSE may arise from diverse aetiologies, including viral infections, rare genetic factors, and autoimmune encephalitis (AIE) (6). Notably, up to 50% of NORSE cases remain cryptogenic (c-NORSE) with no identifiable underlying cause, and these cases are often associated with poorer clinical outcomes (9). Among cases with an identified aetiology, AIE represents a leading cause (9). Mechanistically, AIE can be broadly classified into two major categories (10). The first comprises antibody-mediated disease, in which autoreactive B cells produce pathogenic autoantibodies targeting neuronal surface antigens such as antibodies against N-methyl-D-aspartate (NMDA) receptor and γ-aminobutyric acid (GABA_A_/GABA_B_) receptors, leading to receptor dysfunction and synaptic disruption. The second category involves T cell–mediated disease, in which intracellular neuronal antigens are presented on major histocompatibility complex (MHC) class I molecules and recognised by cytotoxic CD8^+^ T cells, resulting in direct neuronal injury through cell-mediated cytotoxicity. The growing recognition of AIE as a leading identifiable cause of NORSE, underscores the central role of immune dysregulation in disease pathogenesis.

NORSE is a clinical presentation rather than a specific diagnosis. Critically ill NORSE/FIRES patients are often intubated and mechanically ventilated, requiring prolonged intensive care unit (ICU) stays. These patients are at exceptionally high risk of malnutrition due to catabolic stress from ongoing seizures, parenteral/enteral feeding, and altered gastrointestinal function. In addition, sepsis, intercurrent infections, and the development of multiorgan dysfunction can further alter the systemic and central nervous system cytokine responses. Similarly, aggressive therapies, including administration of sedatives, anaesthetic agents, and antiseizure medications, may also influence the immune signatures. Collectively, these factors can independently contribute to elevations in both pro- and anti-inflammatory cytokines. These overlapping pathophysiology mechanisms complicate the interpretation of cytokine profiles in NORSE/FIRES, as these confounding factors may significantly exaggerate or obscure the true inflammatory state in these disorders (8, 11).

While both antibody-mediated and T cell–mediated mechanisms contribute to neuronal dysfunction, increasing evidence highlights a complex interplay between autoreactive B cells, pathogenic autoantibodies, T cell–driven responses, and cytokine-mediated inflammatory networks that sustain seizure activity. These observations emphasise the need to move beyond aetiological classification towards a unified mechanistic framework integrating adaptive immune responses with cytokine signalling. Accordingly, this review focuses on the roles of B cells and autoantibodies, T cell–mediated immunity, and their interactions with pro-inflammatory cytokine networks. In particular, understanding how these immune components influence blood–brain barrier (BBB) integrity, immune cell trafficking, and neuroinflammation will be critical for elucidating disease progression and guiding therapeutic strategies in NORSE.

## 2 Methods

This narrative review was conducted using a non-systematic literature search of PubMed, Scopus, and Web of Science to identify relevant studies published up to 2026. The search focused on literature related to NORSE, B cell, or T cell–mediated immune mechanisms, and cytokine profiling (**Supplementary Table 1**). Original research articles, including cohort studies, case series, and case reports, were considered, with higher priority given to studies providing stronger clinical or mechanistic evidence, such as cohort studies and larger case series. Additional relevant references were identified through manual screening of the reference lists of eligible articles. The included literature was critically evaluated based on study design, sample size, methodological rigour, consistency of findings, and relevance to the review objectives. Given the rarity of NORSE and the limited availability of high-level evidence, findings from well-documented case reports were also incorporated where they provided important mechanistic or clinical insights.

## 3 Pro-inflammatory cytokine networks driving neuroinflammation and BBB disruption in NORSE

In NORSE, markedly elevated levels of serum pro-inflammatory cytokines have been reported, including interleukin 6 (IL-6), IL-12p70, tumour necrosis factor alpha (TNF-α), and key chemokines such as C-X-C motif chemokine ligand 8 (CXCL8/IL-8), C-C motif chemokine ligand 2 (CCL2) and CCL3. Notably, higher concentrations of these cytokines correlate with poorer clinical outcomes (12). These pro-inflammatory mediators converge on shared inflammatory pathways that amplify immune activation and contribute to disease progression in NORSE.

Interestingly, whilst most serum pro-inflammatory cytokines decline over time following SE resolution, c-NORSE patients exhibit persistent or evolving immune activation during the chronic phase, a period of at least 3 months after SE resolution when post-NORSE epilepsy may develop (13, 14). This is characterised by sustained elevated serum levels of adaptive immune disturbances, including IL-12p70, IL-17A, and TNF-α, coinciding with the development of post-NORSE epilepsy (13). Furthermore, dysregulated innate immunity in NORSE, characterised by monocyte–microglial activation and a pro-inflammatory cytokine milieu, is thought to drive BBB dysfunction, antigen presentation and lymphocyte activation, thereby potentially bridging early innate responses with downstream adaptive autoimmunity in susceptible patients (15).

In general epilepsy, IL-6 is associated with promoting neuroinflammation and disrupting blood–brain barrier (BBB) integrity, whilst TNF-α further amplifies inflammatory signalling cascades. In parallel, CXCL8 drives neutrophil recruitment, whereas CCL2 and CCL3 facilitate monocyte recruitment and microglial activation via C-C chemokine receptor type 2 (CCR2) and CCR5, respectively. These coordinated processes enhance immune cell trafficking into the central nervous system (CNS), exacerbating neuroinflammation and tissue injury. Consistent with this, patients with NORSE exhibit increased BBB permeability, particularly in the thalamus and basal ganglia compared with encephalitis patients without SE, thereby permitting leukocyte extravasation into the CNS (16). This heightened and predominantly innate immune activation has been linked to astrogliosis, BBB disruption, and long-term neurological sequelae following NORSE (17).

However, direct causal relationships between individual cytokines, BBB disruption, and immune cell infiltration in NORSE have not been definitively established. It is hypothesised that, in AIE-associated NORSE, acute cytokine storms involving IL-6, TNF-α, CXCL8, and related mediators may promote BBB disruption and permit non-specific (polyclonal) trafficking of peripheral immune cells into the CNS. Following entry into the inflamed CNS, B cells may encounter neuronal surface antigens such as NMDAR, supporting a model in which activation and selection of antigen-specific clones may occur. Within the CNS pro-inflammatory microenvironment, sustained IL-6 signalling may enhance the survival of activated B cells, plasmablast differentiation and clonal expansion, which may be associated with intrathecal antibody synthesis targeting NMDAR.

Persistent inflammatory signalling may facilitate adaptive immune priming by enhancing antigen presentation, promoting T-cell activation, and supporting clonal expansion and long-term survival of autoreactive lymphocyte populations. Following resolution of the acute phase, innate cytokine levels may decline, followed by a transition towards adaptive immune-dominated activity. In particular, IL-12p70, IL-17A, and TNF-α contribute to the maintenance of pro-inflammatory T-cell effector memory phenotypes by promoting T (T_H_) helper cell differentiation, particularly T_H_1 and T_H_17 lineages. These activated T cells may in turn contribute to sustained activation of autoreactive B-cell clones, including NMDAR-specific B-cell responses through cytokine signalling and cell–cell interactions. Collectively, these processes may sustain long-term neuroimmune activation and potentially contribute to post-NORSE epileptogenesis. Nevertheless, there is no definitive evidence establishing the temporal sequence or causal hierarchy of innate and adaptive immune activation in AIE-mediated NORSE or c-NORSE. The proposed mechanistic linkage between these immune compartments therefore remains hypothetical.

## 4 T-cell infiltration and activation in NORSE

Emerging evidence indicates increased T-cell infiltration in the brain of NORSE patients, consistent with the findings reported in epilepsy (15, 16, 18). This is supported by the presence of CSF pleocytosis, with a median CSF white blood cell count of 18/μl, predominantly comprising lymphocytes (15). Such lymphocyte predominance is further corroborated by single-cell transcriptomic data demonstrating T-cell presence exceeding baseline immune surveillance levels in the CNS of NORSE patients (18). Similarly, neuropathological studies in NORSE indicate that 14% of the cases exhibit perivascular leukocyte infiltration within the CNS, predominantly consisting of CD8^+^ T cells (19). This infiltration is likely facilitated by BBB disruption and a concurrent pro-inflammatory milieu that promotes immune cell trafficking into the CNS. From a therapeutic perspective, targeting T cell–mediated inflammation may offer clinical benefit.

### 4.1 CD8^+^ cytotoxic T cell–mediated neuronal injury in NORSE

T cells in the hippocampus of NORSE patients exhibit greater functional activity than those in the cortex, as indicated by gene expression signatures associated with CD8^+^ cytotoxic activity, apoptosis, and immune infiltration (18). Apart from human studies, complementary evidence from animal models reinforces this association, as studies using pilocarpine-induced epilepsy in mice and rats demonstrate increased splenic CD3^+^ and CD8^+^ T-cell populations, highlighting a contributory role of lymphocytes in seizure development (20). Furthermore, perforin, a key cytolytic effector released upon CD8^+^ T-cell activation, has been identified as a critical mediator linking peripheral intravascular inflammation to seizure activity (20), in part through the induction of vascular permeability and BBB disruption (21).

**Figure 1:**
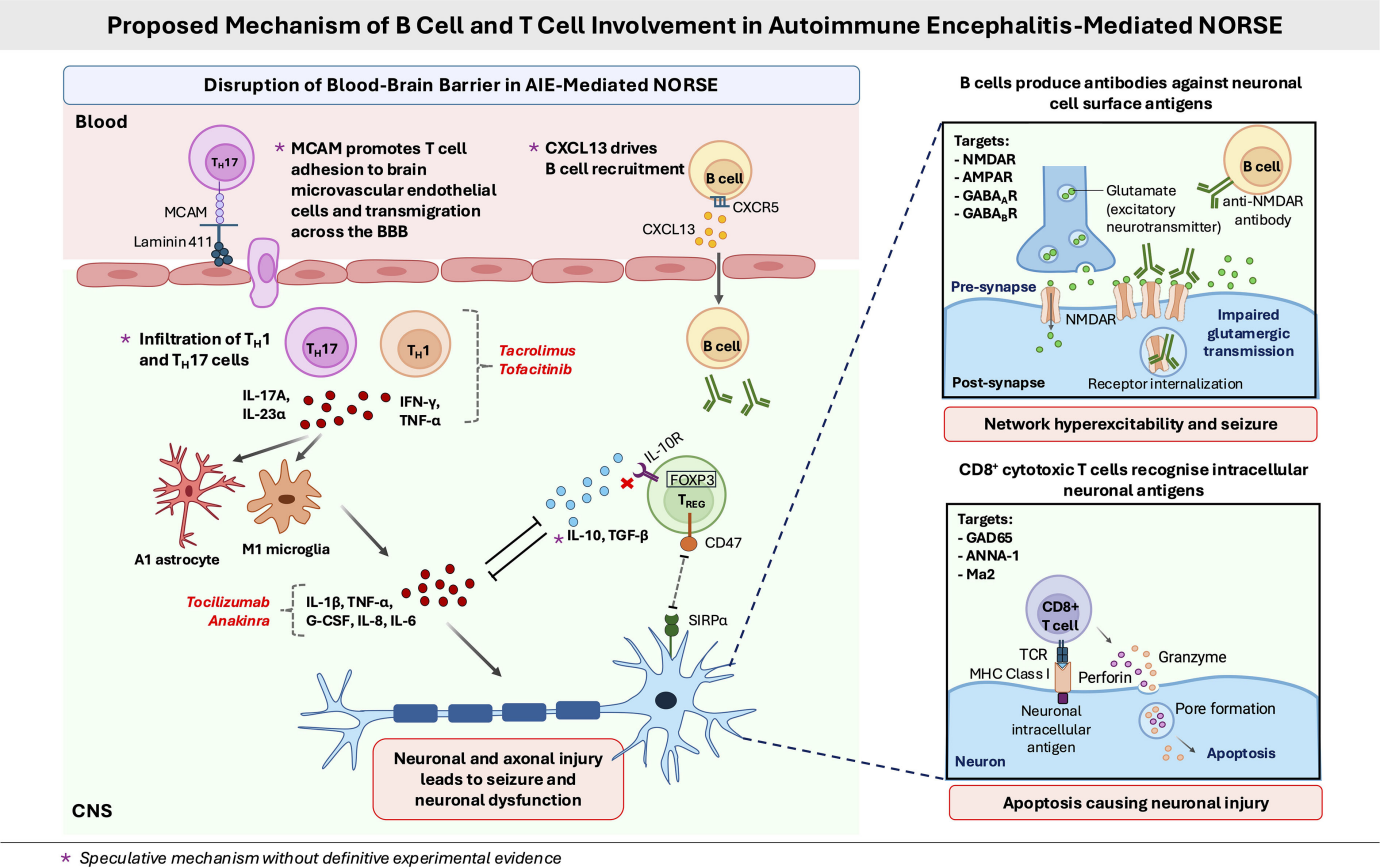
Proposed mechanism of B-cell and T-cell involvement in AIE-mediated NORSE. Disruption of the BBB facilitates infiltration of peripheral immune cells into the CNS. CXCL13 released by stromal cells drives B-cell recruitment whereas MCAM promotes T-cell adhesion to brain microvascular endothelial cells and transmigration across the BBB. Infiltrating T_H_1 and T_H_17 cells release pro-inflammatory cytokines such as IFN-γ, TNF-α, IL-17A, and IL-23a, which promote the pro-inflammatory state of microglia and astrocytes. M1 microglia and A1 astrocytes further amplify oxidative stress and excitotoxicity through pro-inflammatory cytokine production, ultimately leading to neuronal and axonal injury. Meanwhile, excessive pro-inflammatory mediators impair T_REG_ function by disrupting IL-10 signalling, leading to reduced FOXP3 expression. Reduced T_REG_ numbers result in decreased production of anti-inflammatory cytokines such as IL-10 and TGF-β. The neuroprotective effect of T_REG_ via CD47-SIRPα signalling is also diminished. Existing immunotherapies, including tacrolimus and tofacitinib, can inhibit T-cell activation and differentiation, whereas tocilizumab and anakinra reduce pro-inflammatory cytokine signalling. In NORSE secondary to AIE, B cells produce antibodies against neuronal cell-surface antigens such as NMDAR, AMPAR, GABA_A_R, or GABA_B_R, causing receptor internalization and synaptic dysfunction. CD8^+^ cytotoxic T cells recognise intracellular neuronal antigens including GAD65, ANNA-1, or Ma2, and induce neuronal damage via perforin-mediated pore formation and granzyme-dependent apoptosis. Ultimately, these mechanisms lead to neuronal injury, disruption of excitatory–inhibitory synaptic balance, network hyperexcitability, and seizure generation and perpetuation.

CD8^+^ cytotoxic mechanisms are well established in AIE associated with intracellular antigens, such as anti-glutamic acid decarboxylase 65 (GAD65), anti-Hu (anti-neuronal nuclear antibody type 1, ANNA-1), and anti-Ma2 (Paraneoplastic Ma antigen 2, PNMA2) encephalitis (**Figure 1**) (22). These intracellular neuronal antigens serve distinct physiological functions within the CNS. GAD65 catalyzes the conversion of GABA, thereby maintaining excitatory–inhibitory balance. The Hu protein is neuronal RNA-binding protein involved in the regulation of mRNA stability, splicing, and a neuronal differentiation, whereas Ma2 is implicated in neuronal signalling and survival, although its precise role remains incompletely defined.

In these conditions, intracellular neuronal antigens are processed and presented on MHC class I molecules, triggering autoreactive CD8^+^ cytotoxic T-cell responses. Upon activation, these cells induce neuronal injury through perforin-mediated membrane pore formation and granzyme B–dependent activation of caspase pathways, leading to apoptosis. Notably, this cytotoxic process occurs irrespective of the presence of detectable autoantibodies, indicating that neuronal injury is predominantly driven by T cell–mediated mechanisms rather than humoral responses.

Autoantibodies directed against these intracellular neuronal antigens are typically not directly pathogenic but instead reflect an underlying cytotoxic CD8^+^ T cell–mediated immune response. When present, these autoantibodies may exert variable or secondary effects. For instance, GAD65 autoantibodies may contribute to neuronal dysfunction by impairing GABA synthesis and disrupting inhibitory neurotransmission, thereby promoting neuronal hyperexcitability. In contrast, anti-Hu and anti-Ma2 antibodies primarily serve as biomarkers of paraneoplastic encephalitis rather than direct mediators of neuronal injury, as neuronal damage in these conditions is largely driven by CD8^+^ T cell-mediated cytotoxicity targeting antigen-expressing neurons. Collectively, these findings support a model in which CD8^+^ T cell-driven cytotoxicity represents a dominant mechanism of neuronal injury in NORSE, operating largely independently of direct autoantibody-mediated effects.

### 4.2 CD4^+^ T-cell polarisation towards pro-inflammatory T_H_1 and T_H_17 subsets in NORSE

Single-cell RNA sequencing data from the brains of NORSE patients reveals the presence of a CD4^+^ T-cell subtype expressing canonical lineage markers, including *PTPRC (CD45)*, *CD2*, *IL-7R*, and *CD4* (18). However, their precise phenotypic characterisation remains incompletely defined. Available evidence is therefore largely extrapolated from studies in AIE and epilepsy, demonstrating that pro-inflammatory CD4^+^ T_H_ subsets, particularly T_H_1 and T_H_17 cells, play important roles in driving CNS inflammation. Supporting this, flow cytometric analysis of CSF in anti-NMDAR encephalitis demonstrates an increased population of IL-17-producing CD4^+^ T cells (23). In addition, a higher proportion of CD4^+^ T cells expresses T_H_17-related adhesion molecule CD146/melanoma cell adhesion molecule (MCAM) has been observed in patients with epilepsy compared to healthy controls (**Figure 1**) (24). MCAM is known as an activation marker of CD4^+^ T cells and it promotes CNS infiltration of pro-inflammatory T cells by mediating adhesion to laminin 411 expressed on brain microvascular endothelial cells and facilitating transmigration across the BBB (25–27). Meanwhile, interferon-gamma antisense RNA 1 (IFNG-AS1), a marker of the T_H_1 function associating with IFN-γ expression, is overexpressed in the temporal cortex (18), suggesting the activation of T_H_1 cells in NORSE.

Activated CD4^+^ T cells, including T_H_1 and T_H_17 cells produce a range of proinflammatory cytokines that contribute to neuroinflammatory pathology (28). T_H_17 cells, in particular, secrete key pro-inflammatory mediators, including IL-17A, IL-6, granulocyte–macrophage colony-stimulating factor (GM-CSF), and TNF-α. Consistent with this, a significant increase in IFN-γ, TNF-α, and IL-17A, along with IL-7 and IL-15, has been reported in anti-NMDAR encephalitis (29). Furthermore, T_H_17-related cytokines, specifically IL-17 and IL-23, are elevated in serum from the individuals with anti-NMDAR and anti-leucine-rich glioma-inactivated 1 (LGI1) AIE (30). These pro-inflammatory cytokines contribute to neuronal injury by activating glial cells, promoting oxidative stress, and enhancing excitotoxicity (31). IL-17A can directly augment neuronal excitability and indirectly modulate neural function through its effects on glial and immune cells, thereby facilitating the development and progression of epilepsy (32). However, the contribution of T_H_1 and T_H_17 pathways in NORSE secondary to AIE remains uncertain and warrants further investigation, as current evidence is extrapolated from AIE and epilepsy.

### 4.3 T_REG_ depletion weakens anti-inflammatory control in paediatric FIRES

T_REG_ play a central role in limiting neuroinflammation and maintaining immune homeostasis within the CNS. Depletion of T_REG_ has been shown to exacerbate astrocytosis, microgliosis, production of inflammatory cytokines, oxidative stress, and neuronal damage in the hippocampus following SE (33). Consistently, the absence of brain-resident Tregs led to heightened seizure activity and behavioural deficits in experimental models of chronic temporal lobe epilepsy (33). In clinical settings, children with FIRES exhibit naïve CD4^+^ lymphopenia, reduced T_REG_ cell numbers, and weakened phagocytosis, further supporting a loss of immune regulatory capacity (34).

Beyond their suppressive function, T_REG_ actively promote neuroprotection by sustaining anti-inflammatory states in microglia (M2) and astrocytes (A2) by secreting IL-10, IL-4, and TGF-β (35). In addition, T_REG_ enhance the secretion of brain-derived neurotrophic factor (BDNF) from M2 microglia to support neuronal survival and repair (35, 36). Direct neuroprotective effects have also been described through interactions between CD47 on T_REG_ and signal regulatory protein alpha (SIRPα) on neurons, which suppress apoptotic signalling pathways (**Figure 1**) (37). In NORSE, excessive pro-inflammatory mediators may impair T_REG_ function by suppressing prostaglandin E2 (PGE2) synthesis and disrupting IL-10 signalling, leading to reduced Forkhead box P3 (FoxP3) expression and compromised regulatory capacity (38).

While the paediatric FIRES study suggests potential impairment in T_REG_ populations, these findings cannot be confidently extended to adult NORSE. The lack of direct immunophenotyping data in adult cohorts, together with fundamental developmental differences in immune regulation, BBB integrity, and cytokine dynamics, highlights an important gap in current mechanistic understanding and underscores the need for adult-specific immune profiling studies.

## 5 B cell–mediated autoimmunity in the pathogenesis of NORSE

B cells play a critical role in NORSE through the production of pathogenic autoantibodies. Anti-NMDAR encephalitis represents the most common aetiology of NORSE with an identifiable cause (6). These autoantibodies can access the CNS, particularly in the setting of BBB disruption, where they bind to neuronal surface antigens and impair synaptic function, resulting in subsequent neuronal hyperexcitability and seizures. In parallel, BBB disruption also permits the entry of autoreactive B and plasma cells into the CNS to further sustain local autoantibody production and neuroinflammation.

The use of B cell–depleting therapy in NORSE secondary to AIE is supported by both clinical evidence and an improved understanding of the underlying disease mechanisms. A previous case report describes the successful treatment of NORSE secondary to AIE with rituximab in combination with dexamethasone, resulting in marked B-cell depletion and improvement in seizure control (39). This therapeutic approach is supported by evidence from anti-NMDAR encephalitis, where patients exhibit a selective expansion of CD19^+^ B cells in CSF, whilst the T-cell population remains unchanged compared to non-inflammatory neurological controls (40). Furthermore, a neuropathological study further showed substantial accumulation of plasmablasts and CD138^+^ plasma cells within perivascular and parenchymal inflammatory infiltrates in anti-NMDAR encephalitis, highlighting a dominant humoral immune response (41). Interestingly, patients with anti-NMDAR encephalitis show distinct B cell alterations in both CSF and peripheral blood (42). The CSF is enriched with memory B cells exhibiting immune-regulatory gene signatures, whilst peripheral B cells display enhanced antigen presentation profiles. Increased frequencies of IgD^−^CD27^−^ double-negative B cell subsets are observed in circulation, and *in-vitro* studies demonstrate that these cells can differentiate into antibody-secreting cells capable of producing anti-NMDAR autoantibodies. The use of rituximab targets CD20^+^ B cells, effectively depleting the peripheral naïve and memory B-cell compartments as well as short-lived plasmablasts, thereby reducing the production of pathogenic autoantibodies implicated in disease progression (43).

B cell–related chemokines further support a role for humoral immunity in NORSE. C-X-C motif chemokine ligand 13 (CXCL13) levels are elevated in the CSF of patients with FIRES compared to other forms of epilepsy (44). A similar finding is observed in anti-NMDAR encephalitis, where elevated CSF concentrations of CXCL10 and CXCL13 levels have been reported (29). CXCL13 is released by stromal cells in the CNS and acts on CXCR5 expressed on B cells that further drives B-cell recruitment into the CNS, where these cells can differentiate into antibody-secreting plasma cells and contribute to disease pathogenesis (**Figure 1**) (45, 46). Ongoing intrathecal B-cell activation and plasma cell differentiation further sustain the production of pathogenic antibodies, perpetuating receptor dysfunction and seizure activity (41). These findings collectively suggest that B cells may contribute to NORSE through the production of autoantibodies against neuronal antigens, particularly in cases with a suspected autoimmune aetiology.

## 6 Neuronal surface autoantibodies as drivers of NORSE pathogenesis

Neuronal surface autoantibodies directed against key synaptic receptors, including NMDA receptors, α-amino-3-hydroxy-5-methyl-4-isoxazolepropionate (AMPA) receptors, and GABA_A/B_ receptors, represent a major pathogenic mechanism in AIE and are increasingly implicated in NORSE (22, 47, 48) (**Table 1**). In contrast to CD8^+^ T cell–mediated AIE associated with intracellular antigens where neuronal injury is driven by direct cytotoxicity, neuronal surface autoantibody–mediated disease primarily disrupts synaptic function without inducing overt neuronal cell death. This functional, and often reversible, mode of injury distinguishes antibody-mediated mechanisms and underpins the generally more favourable response to immunotherapy (49).

**Table 1:** Autoimmune encephalitis targets, mechanisms, and association with NORSE.

Target	Description	Pathogenic mechanism	Clinical features	Evidence for antibody causing SE/RSE	Evidence specifically in NORSE cohorts	Case reports/series vs. cohort proportion
NMDAR (GluN1)	Ionotropic glutamate receptor located at the synapse; mediates excitatory transmission and synaptic plasticity	Antibody-mediated receptor crosslinking and internalisation, resulting in reduced receptor density and synaptic dysfunction	Psychiatric symptoms, seizures, dyskinesia, autonomic instability	(52, 54, 55)	Strong	Cohort studies (69, 101), Case series (72, 102)
AMPAR (GluA1/2)	Ionotropic glutamate receptor located at the synapse; mediates fast excitatory transmission	Antibody-induced receptor crosslinking and internalisation, leading to reduced synaptic receptor density and impairment of fast excitatory transmission	Limbic encephalitis, memory deficits, seizures; often paraneoplastic	(58–60)	–	–
GABA_A_R	Ionotropic inhibitory receptor located at the synapse; mediates fast inhibitory transmission	Antibody-mediated receptor internalisation or functional blockade, resulting in loss of inhibitory tone and increased neuronal excitability	Severe seizures, encephalopathy, multifocal brain lesions	(64)	–	–
GABA_B_R	Metabotropic inhibitory receptor located at the synapse; modulates neuronal excitability	Antibody-mediated disruption of receptor function, leading to impaired inhibitory signalling	Limbic encephalitis, prominent seizures; often paraneoplastic	(63)	Moderate	Case report (102)
LGI1	Secreted synaptic protein associated with the VGKC complex; regulates AMPAR clustering through ADAM22/23 interactions	Antibody-mediated disruption of protein–protein interactions, leading to altered synaptic signalling	Limbic encephalitis, faciobrachial dystonic seizures, memory impairment	(67, 68)	–	–
CASPR2	Membrane protein associated with the VGKC complex; located at juxtaparanodal regions and involved in potassium channel organisation	Antibody-mediated disruption of potassium channel clustering, resulting in neuronal hyperexcitability	Morvan syndrome, neuromyotonia, with or without encephalitis	(67, 68)	Moderate	Case report (102)

### 6.1 Anti-NMDAR encephalitis as a prototypical autoantibody-mediated driver of NORSE

The NMDA receptor (NMDAR) is a key mediator of synaptic transmission and neuronal excitability, functioning as a tetrameric ion channel composed of two obligatory GluN1 subunits and two GluN2 (A–D) subunits. Anti-NMDAR encephalitis is driven by autoantibodies targeting the GluN1 subunit (48). These antibodies do not primarily induce neuronal death; rather, they bind to the NMDAR surface, causing receptor crosslinking and internalisation, which reduces receptor density and disrupts glutamatergic signalling (50, 51). This synaptic dysfunction leads to an imbalance between excitatory and inhibitory neurotransmission, resulting in network instability that manifests clinically as psychiatric symptoms, cognitive impairment, and seizures (52). In some patients, this progresses to sustained neuronal hyperexcitability and SE, which may evolve into NORSE.

Anti-NMDAR encephalitis is now recognised as one of the most common identifiable autoimmune causes of NORSE, as demonstrated in cohort studies where AIE, particularly anti-NMDAR, accounted for a significant proportion of cases with previously cryptogenic SE (6). Subsequent study further highlighted that autoimmune mechanisms represent a major subset of NORSE and FIRES, with anti-NMDAR encephalitis frequently identified amongst the leading aetiologies (53). Earlier clinical reports also described severe anti-NMDAR encephalitis cases presenting with RSE and prolonged intensive care courses, supporting its role as a prototypical immune-mediated cause of NORSE (54, 55).

Studies have described patients presenting with NORSE who achieved seizure control following early and intensive immunotherapy, including corticosteroids, intravenous immunoglobulin, plasma exchange, and subsequent rituximab. For example, early intensive immunotherapy strategies incorporating rituximab result in favourable neurological outcomes and resolution of RSE in individual NORSE cases, supporting the potential benefit of early aggressive immunomodulation (56). More broadly, observational data in AIE indicate that second-line B cell–depleting therapy such as rituximab is effective in achieving seizure control in patients who fail first-line treatment, with a substantial proportion attaining seizure freedom or significant reduction in seizure burden (57). These findings support the concept that NORSE in anti-NMDAR encephalitis is potentially reversible when the underlying autoimmune process is promptly identified and treated.

### 6.2 Anti-AMPAR encephalitis driving synaptic dysfunction and limbic network hyperexcitability in NORSE

Extending this paradigm, neuronal surface autoantibodies targeting other glutamatergic receptors, such as AMPA receptors (AMPAR), likewise perturb synaptic transmission and drive network instability associated with seizures and NORSE. AIE targeting other synaptic receptors demonstrates both overlapping and distinct pathogenic mechanisms compared to anti-NMDAR encephalitis. Anti-AMPAR encephalitis is classically a form of limbic encephalitis, predominantly affecting the hippocampus and medial temporal lobes (58). Autoantibodies against GluA1 or GluA2 subunits induce receptor internalisation, resulting in reduced synaptic AMPAR density and impaired fast excitatory transmission (58, 59). This disruption compromises network synchrony and promotes compensatory neuronal hyperexcitability. Both anti-NMDAR and anti-AMPAR encephalitis can present with seizures and may progress to SE or NORSE, although anti-AMPAR encephalitis is more frequently associated with limbic features and paraneoplastic processes (60). Although clinical data specific to anti-AMPAR encephalitis remain limited, rituximab is frequently employed as a second-line therapy in refractory cases, with available case reports and observational studies suggesting clinical improvement, consistent with its established efficacy in other forms of AIE (61).

### 6.3 GABAergic dysfunction driving refractory seizures and pharmacoresistance in NORSE

GABAergic dysfunction represents a key mechanism contributing to seizure generation and persistence in NORSE. GABA is the principal inhibitory neurotransmitter in the CNS, acting primarily through GABA_A_ and GABA_B_ receptors to suppress neuronal excitability (62). AIE targeting GABA receptors exhibits subtype-specific patterns of involvement. GABA_B_ receptor encephalitis typically presents as limbic encephalitis, characterised by prominent seizures and memory impairment, and is frequently associated with paraneoplastic conditions (63). In contrast, GABA_A_ receptor encephalitis is associated with more diffuse cortical involvement and is linked to refractory seizures and SE (64).

In both conditions, autoantibodies disrupt receptor function through internalisation or functional blockade, leading to impaired inhibitory synaptic transmission and increased neuronal excitability, consistent with experimental evidence demonstrating antibody-mediated removal of synaptic receptors and consequent network dysfunction. Furthermore, prolonged SE can exacerbate this dysfunction by promoting activity-dependent internalisation and reduced surface expression of GABA_B_ receptors, contributing to pharmacoresistance to GABAergic agents such as benzodiazepines and perpetuating refractory seizure activity (65, 66).

### 6.4 Expanding spectrum of neuronal surface autoantibodies in NORSE

In addition to NMDAR, AMPAR, and GABA receptor antibodies, an expanding spectrum of neuronal surface autoantibodies has been implicated in NORSE and related forms of RSE. These include antibodies against leucine-rich glioma-inactivated 1 (LGI1) and contactin-associated protein-like 2 (CASPR2), which were historically classified under voltage-gated potassium channel (VGKC)–complex antibodies (67, 68). LGI1 antibodies are strongly associated with limbic encephalitis and seizure disorders, whereas CASPR2 antibodies are linked to a broader range of neuroimmune syndromes with variable seizure involvement. Additional, less common targets include dipeptidyl-peptidase-like protein 6 (DPPX), glycine receptor (GlyR), and metabotropic glutamate receptor 5 (mGluR5), all of which have been associated with neuronal hyperexcitability and encephalitic presentations that may include seizures or SE.

Although these autoantibodies are less frequently identified in NORSE compared with anti-NMDAR and anti-GABA_A/B_R encephalitis, their recognition underscores the expanding spectrum of autoimmune-mediated synaptic dysfunction contributing to refractory seizures. Emerging cohort and review data suggest that AIE accounts for a substantial proportion of previously c-NORSE cases, with diverse neuronal surface autoantibodies implicated in disease pathogenesis. These findings indicate that more comprehensive and systematic autoantibody screening may improve diagnostic yield, reclassify a subset of c-NORSE cases as autoimmune-mediated, and facilitate earlier initiation of targeted immunotherapy.

## 7 Comparative features of AIE-associated NORSE and c-NORSE

A single multi-proteomic study comparing anti-NMDAR encephalitis–associated NORSE and c-NORSE has reported distinct pathway enrichment patterns between the two groups (69). In anti-NMDAR encephalitis–associated NORSE, pathways related to humoral immune responses, wound healing, and epigenetic regulation of transcription were upregulated. In contrast, c-NORSE showed enrichment of pathways involved in innate and lymphocyte-mediated immune responses, synaptic function, ubiquitination, and apoptosis. No significant differences in overall inflammatory cytokine levels were observed between the two groups, possibly due to the limited sample size (*n* = 5). Consistent with the proteomic findings, pro-inflammatory cytokines IFN-γ and TNF-α, as well as anti-inflammatory cytokines IL-4 and IL-13, were increased in anti-NMDAR encephalitis–associated NORSE, whereas IL-6 and IL-8 were elevated in c-NORSE (69).

Current evidence of this review is more heavily focused on AIE-mediated NORSE than on c-NORSE, and the exact mechanisms underlying c-NORSE remain largely unknown. **Table 2** compares whether the mechanisms discussed above share similarities with c-NORSE and which aspects may be cautiously extrapolated versus those that are likely distinct.

**Table 2:** Comparative proposed immune mechanisms in animal model, AIE-associated NORSE and c-NORSE cases.

Immune component	NORSE	AIE-associated NORSE	c-NORSE	Interpretation
Innate immune activation (microglia, monocytes, cytokines)	Commonly reported in cytokine profiling cohort studies	Upregulated of pro-inflammatory cytokines were reported	Predominant inflammatory signature was suggested	The relative hierarchy and upstream drivers of these immune pathways remain unresolved
Level of evidence	Animal study (103) Cohort studies (12, 101)	Case series (101, 102)	Cohort studies (12, 70)	
B cells/antibodies	-	Pathogenic autoantibodies (e.g. NMDAR, GAD65) were identified	Usually seronegative; pathogenic antibodies were not identified	Strong antibody-mediated mechanism in AIE; down regulated in c-NORSE
Level of evidence	–	Animal study (69) Case series (72, 101, 102)	Cohort study (69) Case series (70)	
CD8+ T cells	-	May contribute to neuronal injury via cytotoxic mechanisms in some AIE subtypes (e.g., anti-Hu, Ma2, GAD65-associated encephalitis)	Cytotoxic effector activity is hypothesised based on elevated granzyme B level, but their contribution to neuroaxonal injury and inflammatory signalling remains unclear.	Evidence stronger in AIE; speculative but potentially important in c-NORSE
Level of evidence	–	Case report (101)	Cohort study (70)	
CD4+ T cells	-	Polarisation towards pro-inflammatory T_H_1/T_H_17 phenotypes contribute to cytokine-mediated neuroinflammation and BBB dysfunction	May involve in downstream adaptive immune activation, contributing to post-NORSE epilepsy	Temporal sequence is not well defined in c-NORSE
Level of evidence	–	Cohort study (69)	Cohort study (13)	

Elevated serum levels of CXCL8 and CCL2 and CCL3 reported in c-NORSE compared with NORSE of known aetiology, may indicate a predominantly innate immune-driven inflammatory process (12, 70). At the molecular level, a subset of c-NORSE patients demonstrates upregulation of JAK-STAT signalling pathways in serum sample (70). As a central mediator of cytokine signalling across multiple immune cell types, the JAK-STAT pathway drives broad immune activation, including the regulation of T-cell differentiation and effector function. Therapeutically, this provides a rationale for targeting dysregulated cytokine signalling and downstream immune responses. Tofacitinib, a JAK inhibitor, has shown benefit in some cryptogenic FIRES and AIE cases (71, 72). The contribution of CD8^+^ T cells in c-NORSE is further supported by elevated serum granzyme B level, a marker of cytotoxic T-cell activity, compared with patients with RSE of known aetiology, supporting a likely immune-mediated component (70).

A recent study has suggested c-NORSE patients exhibit persistent or evolving immune activation during the chronic phase (13). This is characterised by sustained elevated serum levels of IL-12p70, IL-17A, and TNF-α, coinciding with the development of post-NORSE epilepsy. Sustained IL-12p70 signalling may promote T_H_1 subset differentiation and the formation of long-lived inflammatory memory T-cell subsets through STAT4-dependent pathways, whereas upregulated IL-17A and TNF-α may suggest involvement of the T_H_17 axis. While both innate and adaptive immune responses are implicated in c-NORSE, the directionality and temporal sequence of their interaction remain undefined.

## 8 Age- and sex-related considerations in NORSE

Age and sex are important biological variables that may influence immune responses in NORSE. FIRES is defined as a clinical subtype of NORSE characterised by the occurrence of a preceding febrile illness, with the absence of an identified acute infectious cause. Although FIRES is predominantly reported in paediatric populations, this reflects epidemiological distribution rather than a strictly age-defined biological entity. In contrast, adult NORSE is more heterogeneous with respect to underlying aetiology, clinical presentation, and immune profiles.

Age-related differences in immune function may contribute to variability in disease expression. The developing immune system in children is characterised by ongoing maturation of adaptive immunity, increased innate immune responsiveness, and differences in BBB integrity and microglial activation compared with adults. These factors may influence susceptibility to sustained neuroinflammation and epileptogenesis. Furthermore, the direct comparative mechanistic studies between paediatric and adult NORSE populations remain limited.

Sex-related differences are well established in autoimmune conditions, particularly in antibody-mediated disorders such as anti-NMDAR encephalitis, which demonstrates a marked female predominance and recognised paraneoplastic associations (57). These differences are thought to be influenced by sex hormones and X-linked immune regulatory genes, which modulate B-cell activity, T-cell polarisation, and cytokine responses.

Overall, the current lack of robust age- and sex-stratified immunological data in NORSE represents an important limitation. FIRES may reflect a mechanistically distinct entity from adult autoimmune NORSE, and therefore direct extrapolation should be interpreted carefully. Future studies incorporating stratified immunological profiling are required to determine whether clinically relevant age- or sex-associated immune endotypes exist in NORSE.

## 9 Future horizons of targeted cytokine and T_H_17-directed immunotherapies in NORSE

### 9.1 Emerging mechanism-based immunotherapy strategies in NORSE

Immunotherapy remains the cornerstone of current treatment for c-NORSE. First-line immunotherapy typically includes corticosteroids, intravenous immunoglobulin (IVIG), or plasmapheresis (73). In patients who do not respond to initial treatment, second-line immunotherapy such as cyclophosphamide and/or rituximab is commonly employed (74). For treatment-refractory cases, alternative therapeutic strategies such as tocilizumab (IL-6 receptor antagonists) and anakinra (IL-1 receptor antagonists) have been employed to mitigate excessive pro-inflammatory cytokine-driven neuroinflammation within the CNS (75–78).

Despite these advances, a substantial proportion of patients with c-NORSE remain refractory to current immunotherapeutic approaches, underscoring critical gaps in our understanding of the underlying immunopathology. The marked heterogeneity of immune mechanisms implicated in NORSE, including antibody-mediated synaptic dysfunction, CD8^+^ T cell-driven cytotoxicity, and dysregulated CD4^+^ T_H_ responses, suggests that a single-modality treatment strategy is unlikely to be sufficient.

As discussed above, emerging evidence supports the presence of distinct immunological endotypes underlying NORSE subgroups, characterised by either B cell- and autoantibody-driven mechanisms or T cell– and innate immune-mediated neuroinflammation. Accordingly, more refined immunophenotyping and biomarker-guided stratification are needed to inform targeted therapeutic interventions. For example, B cell–depleting therapies such as rituximab may be particularly effective in antibody-mediated disease, whereas T cell–directed approaches, including calcineurin inhibitors or Janus kinase–signal transducer and activator of transcription (JAK–STAT) pathway inhibitors, may be more appropriate in cases characterised by cellular immune activation. Likewise, cytokine-targeted therapies such as tocilizumab and anakinra illustrate the therapeutic potential of modulating upstream inflammatory networks.

Therefore, these observations highlight the need for a shift towards a mechanism-based immunotherapy framework that integrates modulation of B cell, T cell, and cytokine pathways. Future therapeutic strategies may include combinatorial immunomodulation and the development of novel agents targeting specific immune axes, such as selective cytokine blockade and cellular therapies, to more effectively control neuroinflammation and improve outcomes in NORSE.

### 9.2 Targeting T_H_1/T_H_17 Axis as a therapeutic strategy in NORSE

**Figure 2:**
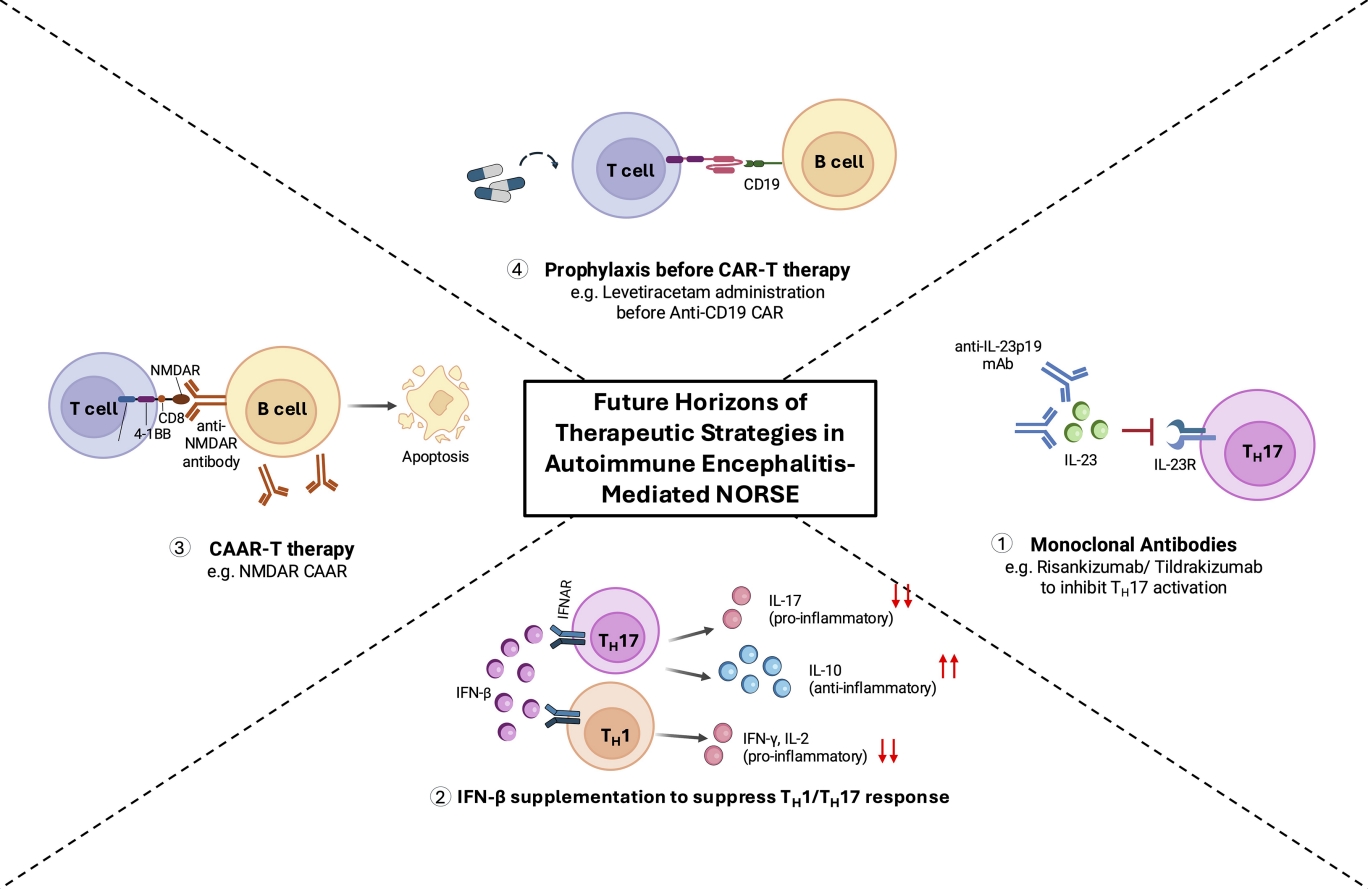
Potential therapeutic strategies in AIE-mediated NORSE. (1) Monoclonal anti-IL-23 antibody therapy, such as Risankizumab and Tildrakizumab, may reduce the overproduction of T_H_17 cells in AIE-mediated NORSE and subsequently inhibit IL-17 production. (2) IFN-β signalling via the type I interferon receptor (IFNAR) may suppress pro-inflammatory T_H_1 and T_H_17 responses and reduce downstream inflammatory cytokine production. (3) NMDAR-Chimeric Autoantibody Receptor (NMDAR-CAAR) T-cell therapy binds specifically to anti-NMDAR-reactive B cell, inducing apoptosis and reducing pathogenic autoantibody production whilst minimising broad immunosuppression. (4) Administration of antiseizure medications as seizure prophylaxis prior to CAR-T cell therapy may help prevent cytokine-induced neurotoxicity.

Given the emerging role of T_H_17-driven neuroinflammation in immune-mediated NORSE, therapeutic strategies targeting the T_H_17 axis represent a promising avenue for intervention. IL-23 is a key survival and maintenance factor for T_H_17 cells, sustaining their pathogenic potential in neuroinflammatory conditions (79, 80). Accordingly, IL-23 blockade using anti-IL-23p19 monoclonal antibodies such as risankizumab and tildrakizumab may represent. promising therapeutic strategy (**Figure 2**). Notably, these antibodies have shown favourable safety profiles in psoriasis (81, 82), and with no reported neurological adverse effects in epileptic patient treated for psoriasis and eczema (83).

Current consensus driven treatment for NORSE includes cytokine-targeted therapies such as tocilizumab (IL-6 receptor antagonist) and anakinra (IL-1 receptor antagonist), which are typically employed as second-line immunotherapies (3). As IL-6 and IL-1β are critical cytokines that initiate and amplify T_H_17 differentiation, IL-23 blockade may act synergistically with these agents to more effectively suppress T_H_17-driven inflammation in NORSE.

In parallel, modulation of both T_H_1 and T_H_17 responses represents another potential therapeutic strategy. Interferon-β (IFN-β), a first-line treatment for multiple sclerosis (84), exerts immunomodulatory effects by inducing the secretion of the immunosuppressive cytokine IL-27 from antigen-presenting cells to promote IL-10 production and inhibit T_H_17 cell activity (85, 86). IFN-β also directly suppresses T_H_1 cell proliferation and reduces their capacity to generate IFN-γ and IL-2 (87). Beyond these effects, IFN-β attenuates T-cell activation, adhesion, and penetration across BBB, thereby limiting T cell–mediated pathogenesis in the CNS (88).

Although T_H_17-associated cytokine pathways have been implicated in several autoimmune and neuroinflammatory disorders, direct evidence demonstrating T_H_17-cell predominance or supporting IL-17/IL-23–targeted therapy in human NORSE remains lacking; therefore, these mechanisms should currently be regarded as exploratory and hypothesis-generating rather than clinically validated therapeutic targets.

## 10 Future horizons of cell-based immunotherapy strategies in NORSE

### 10.1 CAR-T strategies for targeting of autoreactive b cells in NORSE

Chimeric antigen receptor (CAR) T-cell therapy has emerged as a highly promising cancer immunotherapeutic strategy, demonstrating remarkable efficacy in the treatment of refractory hematological malignancies (89, 90). It is an adoptive cellular therapy in which a patient’s T cells are genetically engineered to express a synthetic receptor composed of an extracellular antigen-binding domain derived from a monoclonal antibody fused to intracellular T-cell signalling domains. This chimeric structure enables direct recognition of specific target antigens on diseased cells, thereby bypassing antigen presentation requirements and overcoming MHC restriction. The engineered T cells are expanded *ex vivo* and reinfused to mediate potent and sustained cytotoxicity against target cells.

Conventional B cell–targeted therapies such as rituximab, as well as CD19-directed CAR-T cell approaches, achieve broad depletion of B-cell populations, including both pathogenic and non-pathogenic subsets. While effective in reducing autoantibody production, these strategies are associated with global immunosuppression and an increased risk of infection due to the loss of protective humoral immunity. Interestingly, in 2023, an NMDAR-specific chimeric autoantibody receptor (NMDAR-CAAR) T-cell approach was developed to selectively target B cells producing NMDAR autoantibodies (91). NMDAR-specific CAAR T cells are engineered to express a chimeric receptor comprising an extracellular multi-subunit NMDAR autoantigen fused to intracellular 4-1BB and CD3ζ signalling domains, thereby enabling selective recognition of anti-NMDAR B cell receptors and activation of cytotoxic effector functions (91). Current evidence from preclinical *in-vivo* mouse models demonstrates that NMDAR-CAAR T cells can selectively deplete anti-NMDAR antibody-secreting B cells and reduce autoantibody titres without detectable off-target toxicity (91). This approach represents a promising alternative to conventional CAR-T treatment by avoiding broad immunosuppression; however, to date, human clinical trial have not yet commenced.

### 10.2 Neurotoxicity and seizure risk associated with CAR-T cell therapy

CAR-T cell therapy may potentially cause complications such as cytokine release syndrome (CRS) and immune effector cell-associated neurotoxicity syndrome (ICANS) (92). The risk of developing seizures after CAR-T cell therapy is notably high, reaching up to 30% within the first week post-treatment (93). Seizures and generalised periodic discharges following CAR-T cell therapy are likely driven by elevated cytokine levels that induce thalamocortical hyperexcitability (94). A case study reports that a 22-year-old woman developed nonconvulsive status epilepticus 18 days after receiving axicabtagene ciloleucel, an FDA-approved anti-CD19 CAR-T cell therapy for relapsed or refractory diffuse large B cell lymphoma (95).

To prevent seizure development after CAR-T cell therapy, prophylactic administration of anti-seizure medications was suggested (96). The safety and effectiveness of levetiracetam as a primary or preemptive prophylactic measure in haematological patients undergoing anti-CD19 CAR-T cell therapy were reported (97). In particular, levetiracetam has low cardiotoxicity, minimal drug interactions, and a lower impact on cytokine levels, supporting its safety in CAR-T-cell recipients (98, 99). These findings highlight the potential role of seizure prophylaxis prior to CAR-T cell therapy in clinical settings to prevent the adverse effects.

Additionally, although CAR-T cell therapy offers personalised treatment approach, its production is costly, and variability in cellular stability, limited *ex-vivo* expansion, and *in-vivo* persistence across individuals may contribute to inconsistent clinical outcomes (100). CAR-T cell manufacturing takes several weeks, which may not be suitable for treating the acute phase of NORSE but may have potential utility in the chronic phase to reduce post-NORSE epilepsy. This remains purely hypothetical, with no reported evidence in NORSE to date. Further investigation is required to balance potential benefits against adverse effects before clinical application.

## 11 Future perspectives in NORSE research

Future research should move beyond conventional immune profiling to address the existing knowledge gap regarding the interaction between adaptive immune cells and the neuronal microenvironment in NORSE. In particular, detailed characterisation of T-cell subset activation and functional phenotypes, such as T_H_1 and T_H_17, using approaches such as flow cytometry and cytokine profiling is essential to clarify their contributions to NORSE pathogenesis. Additionally, immune repertoire profiling of T-cell receptor (TCR) and B cell receptor (BCR) clonality and antigen specificity may help determine whether antigen-driven immune responses contribute to disease initiation in c-NORSE. Integrative multi-omics strategies combining single-cell RNA sequencing, spatial transcriptomics, and proteomic profiling of both CSF and serum, as well as brain tissue, may further enable the identification of pathogenic immune cell populations and inflammatory pathways that drive seizure activity in NORSE. Combining these data with clinical, neuroimaging, and electrophysiological recordings could help define how immune cells interact with neurons, astrocytes, and microglia within epileptogenic brain regions and how immune-mediated inflammation alters neuronal networks to sustain seizure activity.

Further investigation into BBB integrity and dysfunction also represents a key priority in neuroinflammatory mechanisms in NORSE. Determining how BBB disruption facilitates immune cell trafficking into the CNS to sustain and amplify neuroinflammation may provide important insights into disease progression and seizure persistence. Advanced imaging modalities together with circulating and CSF biomarkers of endothelial injury may provide insights into the timing and mechanisms of immune cell trafficking during NORSE. Therapeutic strategies aimed at restoring BBB integrity in NORSE, including approaches that stabilise endothelial tight junctions or inhibit matrix metalloproteinase–mediated barrier breakdown, may limit immune cell entry into the CNS and mitigate sustained neuroinflammatory responses.

A critical emerging research direction involves the discovery of novel neuronal autoantigens. While known autoantibodies such as anti-NMDAR explain a subset of NORSE cases associated with AIE, many patients remain seronegative for established neuronal antibodies. Advanced techniques such as high-throughput autoantibody discovery platforms and proteomic antigen mapping may help identify previously unrecognised autoantigens that trigger pathogenic immune responses in NORSE.

Finally, the identification of predictive immune biomarkers capable of guiding immunotherapy represents an important translational goal. Longitudinal immune profiling integrated with clinical and neuroimaging data may reveal immune signatures that predict treatment response to targeted therapies such as IL-1 blockade, IL-6 inhibitors, or B-cell–depleting agents. Prospective multicentre studies with larger patient cohorts are essential to validate the prognostic role of immune biomarkers and enable more precise and personalised immunomodulatory strategies for NORSE patients. Although emerging studies suggest that CSF cytokine profiles, autoantibody signatures, and immune-cell phenotypes may eventually facilitate biomarker-guided immunotherapeutic stratification in NORSE, current evidence remains insufficient to support a standardised bedside treatment algorithm, and these approaches should presently be considered exploratory and hypothesis-generating rather than clinically established.

## 12 Conclusion

This review highlights the potential contribution of adaptive immune dysregulation in the pathogenesis of NORSE, although the underlying immune mechanisms remain incompletely understood due to the scarcity of NORSE-specific immunological and mechanistic data. A substantial proportion of the discussion relies on extrapolation from AIE and broader neuroinflammatory disease models, which may not fully capture the pathobiology of NORSE but nevertheless provide useful mechanistic insight. In particular, the temporal and hierarchical sequence of innate and adaptive immune activation in both AIE-mediated NORSE and c-NORSE has not been definitively established. Most available evidence is derived from cross-sectional cytokine profiling and clinical response to immunotherapy rather than longitudinal immune trajectory data. Finally, most advanced therapeutic strategies discussed, particularly cellular therapies such as CAR-based approaches, remain preclinical or experimental in the context of NORSE, with no direct clinical evidence supporting their use. Accordingly, these therapeutic concepts should be considered hypothesis-generating rather than clinically applicable at present. Further elucidation of immune cell subsets and their associated cytokine networks may reveal novel therapeutic targets. Given their central role in disease pathogenesis, immunomodulatory strategies targeting B and T cell–mediated immune responses represent promising approaches to improve clinical outcomes in NORSE patients.
