# Supplementary material for: Adaptive immune dysregulation drives new-onset refractory status epilepticus
Source: Front Immunol. 2026 Jul 30;17:1874730. doi: 10.3389/fimmu.2026.1874730 (PMC13470194; doi:10.3389/fimmu.2026.1874730)
Supplement: Supplementary file 1 [file DataSheet1.docx]

**Glossary**

| Term | Definition |
| --- | --- |
| Adaptive immunity | Antigen-specific immune response mediated by T and B lymphocytes that generates immunological memory |
| Antigen-presenting cell (APC) | Cell (e.g. dendritic cell, macrophage) that presents antigen via major histocompatibility complex (MHC) molecules to activate T cells |
| Autoimmune encephalitis (AIE) | Immune-mediated inflammation of the brain caused by autoantibodies or autoreactive immune cells |
| Blood-brain barrier (BBB) | Specialised endothelial barrier that regulates movement of cells and molecules between blood and the central nervous system |
| CD4⁺ helper T (T_H_) cell | A subtype of T lymphocyte that recognises antigens presented by MHC class II molecules and coordinates adaptive immune responses by secreting cytokines |
| CD8⁺ T cell | Cytotoxic T lymphocyte capable of directly killing target cells |
| Chemokine | Small signalling protein that directs migration of immune cells |
| Cytokine | Soluble signalling protein that regulates immune and inflammatory responses |
| Effector T cell | Activated T cell that performs immune functions such as cytokine secretion or cytotoxicity |
| Innate immunity | Immediate, non-specific immune response involving cells such as neutrophils, macrophages and microglia |
| Interleukin (IL) | A group of cytokines that act as signalling molecules to coordinate communication between immune cells and regulate inflammatory and immune responses |
| Major histocompatibility complex (MHC) | Cell surface molecules that present peptide antigens to T cells |
| Microglia | Resident immune cells of the CNS that mediate immune surveillance and inflammatory responses |
| Neuroinflammation | Inflammatory response occurring within the CNS involving resident and infiltrating immune cells |
| New-onset refractory status epilepticus (NORSE) | Clinical presentation of refractory status epilepticus without a clear acute or active structural, toxic, or metabolic cause in a person without active epilepsy or another relevant pre-existing neurological disorder |
| Regulatory T cell (T_REG_) | CD4⁺ T cell subset that suppresses excessive immune responses and maintains immune tolerance |
| T cell receptor (TCR) | Antigen receptor on T lymphocytes that recognises peptide-MHC complexes |
| T helper 1 (T_H_1) cell | CD4⁺ T cell subset producing pro-inflammatory cytokine such as IFN-γ |
| T helper 17 (T_H_17) cell | CD4⁺ T cell subset producing pro-inflammatory cytokine such as IL-17A |
