# Supplementary material for: Adaptive immune dysregulation drives new-onset refractory status epilepticus
Source: Front Immunol. 2026 Jul 30;17:1874730. doi: 10.3389/fimmu.2026.1874730 (PMC13470194; doi:10.3389/fimmu.2026.1874730)
Supplement: Supplementary file 2 [file Table1.docx]

Table S1. Literature Search Strategy and Study Selection Criteria

| Item | Description |
| --- | --- |
| Databases searched | PubMed, Scopus, and Web of Science |
| Search period | From database inception to 2026 |
| Search terms | *("new-onset refractory status epilepticus" OR NORSE OR "febrile infection-related epilepsy syndrome" OR FIRES) AND ("B cell*" OR "T cell*" OR lymphocyte* OR autoimmun* OR neuroinflamm* OR cytokine* OR interleukin* OR chemokine* OR autoantibod* OR "immune mechanism*" OR immunopathogenesis OR immunotherap*)* |
| Types of studies considered | Original research articles, including cohort studies, case-control studies, case series, and case reports |
| Eligibility criteria | Studies investigating the immunopathogenesis, immune cell mechanisms, cytokine profiling, or immunotherapy in patients with NORSE or related autoimmune encephalitis. Articles published in English were considered. |
| Study selection | Studies were selected based on relevance to the review objectives, with greater emphasis placed on cohort studies and larger case series providing stronger clinical or mechanistic evidence. Well-documented case reports were included when they offered important mechanistic or clinical insights. |
| Additional sources | Reference lists of eligible articles were manually screened to identify additional relevant publications. |
